# Association between adherence to a home exercise program and central sensitization, pain intensity, and functionality in individuals with knee osteoarthritis

**DOI:** 10.1186/s12891-022-05959-6

**Published:** 2022-11-17

**Authors:** Gabriela Nascimento de Santana, Almir Vieira Dibai-Filho, José Edson França da Silva Júnior, Aron Charles Barbosa da Silva, Sulamizia Filomena Costa de Jesus, Patrícia Gabrielle dos Santos, Cid André Fidelis-de-Paula-Gomes

**Affiliations:** 1 Postgraduate Program in Rehabilitation Sciences, Nove de Julho University, Rua Vergueiro, 235/249, Liberdade, CEP, São Paulo, SP 01504-001 Brazil; 2 Postgraduate Program in Physical Education, Universidade Federal do Maranhão, São Luís, MA Brazil

**Keywords:** Pain measurement, Chronic pain, Exercise, Knee osteoarthritis

## Abstract

**Objective:**

To analyze the association between adherence to a home exercise program and central sensitization, pain intensity, and functionality in individuals with knee osteoarthritis (KOA).

**Methods:**

A cross-sectional study was conducted involving 57 individuals with KOA. Evaluations were performed using the Exercise Adherence Rating Scale (EARS), the numerical rating scale (NRPS), the International Knee Documentation Committee (IKDC), The Central Sensitization Inventory (CSI), and the 30 sec sit and stand test (30SSST). Histograms were created to determine the normality of the data. The Kolmogorov-Smirnov test was used to determine the normality of the data. Thus, Pearson’s (r) and determination (R^2^) correlation coefficients were calculated to determine the strength of associations between variables.

**Results:**

No significant association was found between adherence behavior or reasons for adherence and central sensitization symptoms, the intensity of pain on rest and movement, knee disability symptoms, and functionality.

**Conclusion:**

No significant association was identified between adherence to a home exercise program and central sensitization, pain intensity, and functionality in individuals with KOA.

## Introduction

In terms of the clinical management of osteoarthritis of the knee (KOA), well-established evidence supports the use of exercise therapy as a first-line therapeutic intervention [1]. Exercise therapy is characterized as a therapeutic approach based on repeated and regular activities to increase resistance and/or minimize the duration of impairment, correcting and/or improving movement related to functional deficits and deficiencies linked to diseases or injuries. In the case of individuals with KOA, aquatic and/or terrestrial therapeutic exercise programs appear to be safe and effective [1–4]. Should be performed for 8 to 12 weeks, 3 to 5 sessions per week, lasting 1 hour for each session. With an emphasis on exercise programs that involve Pilates, aerobic, and strengthening exercises in their composition. Due to exposure to potentially destabilizing loads and movements, the implementation of exercise programs allows adaptation of the neuromuscular system to conditions that may induce joint instability, especially during functional activities. Resulting in positive effects in improving pain, strength, function, and quality of life, both in the short and long term [2, 3].

In addition to these characteristics, exercise programs can be implemented through home exercises. Performed with or without on-site supervision, they are characterized as inexpensive and require little or no equipment to be performed. Home exercise programs are often provided to patients as part of or an alternative to rehabilitation performed in the clinical setting. Its primary objectives are to enhance results achieved in the clinical environment and/or assist and expand the self-management of long-term health conditions [5–7]. In 1 year of follow-up, in relation to the function of the lower limbs, they present effects almost equal to the exercise programs performed in the clinical environment. Demonstrating that they can improve strength, reduce pain intensity, and improve function in individuals with KOA [4, 5].

However, it is also known that individuals affected by chronic health conditions tend to avoid exercise [4]. Specifically, in relation to KOA, low adherence rates are reported, between 27 to 64% [8]. One of the great challenges for therapists is to maintain the adherence of individuals with chronic pain throughout the performance of exercise programs [9]. Mainly, there are growing concerns about adherence to home exercise programs [4]. In the case of interventions related to rehabilitation, in the medium and long term, the rate of adherence to home exercise programs can be low. It may limit the effectiveness of rehabilitation in terms of pain, function, and probable protection against the recurrence of injuries. And above all, it can reduce patients’ ability to self-manage their health problems [4, 5].

Understanding this context, adherence is defined as the extent to which an individual’s behavior corresponds to the recommendations agreed upon by health professionals [10]. It can be influenced by internal factors (knowledge, understanding, beliefs, self-efficacy about the exercises) and external factors (support, access, climate) [10, 11]. Two systematic reviews [10, 12] concluded that supervised or individualized exercise therapy and self-management techniques can increase adherence to exercise programs. However, the heterogeneity of the analyzed studies made it impossible to isolate or define the specific effects of adherence strategies [10, 12]. Specifically, when it comes to home exercises, adherence seems to be related to the feeling of obligation towards the therapist, the desire to avoid medication, and, over time, the availability of time to exercise [13].

There is a notable lack of analysis related to the impact of chronic pain and functionality variables on adherence to exercise programs for individuals with KOA. There is thus clear uncertainty about how best to help people with KOA adhere to exercise [14]. Especially in the context of home exercise programs. However, before testing interventions or treatment plans. It is necessary to have a better understanding of the impact of different variables linked to chronic pain and functionality on adherence to home exercise programs in individuals with KOA.

Therefore, the aim of this study was to analyze the association between adherence to a home exercise program and central sensitization, pain intensity, and functionality in individuals with KOA. The hypothesis tested is that there is a positive association between levels of adherence to a home exercise program and variables related to chronic pain and functionality in individuals with KOA.

## Methods

### Study design and ethical considerations

A cross-sectional study was carried out between the months of August to December 2021 in accordance with the STrengthening the Reporting of Observational studies in Epidemiology (STROBE Statement) [15]. This type of study was chosen due to the logistical possibility and feasibility of the moment of carrying out the study. Since it was carried out with a care group for individuals with chronic musculoskeletal pain started in 2020 with the beginning of the CoVID-19 pandemic, caused by SARS-CoV 2. In this way, a home exercise program was carried out three times a week, consisting of the following exercises: Isometric contractions of the quadriceps, Supine straight-leg lifts, Leg lifts in the prone position, Passive knee flexion, Passive knee extension, Resistance knee extension, Resistance knee flexion, Shifting the center of gravity (left and right), Shifting the center of gravity (forwards and backwards) [16]. This exercise program was chosen because it is an inexpensive, easy-to-use, safe, and suitable program to be practiced at home. And for presenting positive results in terms of reducing pain intensity and joint stiffness, increasing muscle strength of the lower limbs, and improving balance, mobility, and quality of life [16].

Two physiotherapists were responsible for demonstrating the procedures to be performed in the home exercise program. Each physical therapist had a minimum of 8 years of experience managing interventions for chronic musculoskeletal pain. In this way, the demonstration and training of these procedures took place through video conference. There were no face-to-face meetings to demonstrate and carry out training and/or procedures to be carried out by the home exercise program.

Three physical therapists with an average training time of 8 years were responsible for the stages of carrying out the research. A physical therapist was responsible for subject recruitment and verifying the inclusion and exclusion criteria. Another was responsible for applying the assessment instruments. A third party was responsible for processing and analyzing the data.

Research participants were recruited by verbal invitation, from a list of participants in the care group for individuals with chronic musculoskeletal pain in the city of São Paulo (SP, Brazil). All participants signed an informed consent form. This study was approved by the Research Ethics Committee of Universidade Nove de Julho (n° 47,658,721.9.0000.5511).

### Participants

The sample size was calculated using G*Power 3.1 software. The detection of a moderate association (*r* = 0.50) among the variables was considered for the calculation of the sample size [17]. A statistical power of 80% and alpha of 5% were set. Thus, a minimum number of 30 volunteers was determined.

Participants of both genders, aged between 60 and 80 years [18, 19], diagnosed with KOA, and who performed home exercises at least three times a week in the last 3 months prior to the start of the study were included. The diagnosis of KOA was defined according to the medical history and should include all criteria: knee pain and/or altered function lasting 12 weeks or longer [18, 19], morning stiffness < 30 minutes, pain intensity ≥3, radiographic confirmation, and Grades 2 or 3 of the Kellgren-Lawrence Classification [18, 19]. The diagnosis of KOA was determined through an examination and the written opinion of a specialist in rheumatic diseases, with more than 10 years of experience. This same specialist analyzed the following exclusion criteria adopted: history of knee trauma and/or hip or knee replacement surgery, cognitive impairment, psychological disorder, neurological (sensory or motor) disorder, cancer, severe comorbidities of the heart, liver, and/or kidney, serious psychiatric diseases, systemic, autoimmune, or inflammatory any acute adverse health condition, lameness, and the use of a gait-assistance device.

### Assessments

The Exercise Adherence Rating Scale (EARS) is a self-report instrument developed to assess adherence to home exercise [20]. Translated and cross-culturally adapted into Brazilian Portuguese, it is structured in three sections (A, B, and C) [21]; sections A and C are characterized as optional. For this study, sections B and C were applied, respectively, related to adherence behavior and reasons for adherence to exercise. Section B comprises six items and section C comprises ten items, scored using an ordinal response scale (0 = strongly agree to 4 = strongly disagree). Higher scores indicate greater adherence. A final score of 17/24 can be used as a cut-off point to indicate acceptable adherence behavior [21].

The numerical rating scale (NRPS) is a validated scale for the Portuguese language to assess pain intensity [22]. Characterized by a sequence of numbers, from 0 to 10, in which the value 0 indicates “no pain” and 10 indicates “worst pain imaginable” [22]. The intensity of the assessment was predicted (NRPS-R), based on the previous 7 days, and following movement (NRPS-M), after sitting and standing up from a chair for five repetitions.

The International Knee Documentation Committee (IKDC) is an instrument for evaluating KOA-related symptoms and disabilities. Considered valid and reproducible to be used in the Brazilian population, it consists of 18 items, distributed into three domains: 1. Symptoms – with seven items, including pain, swelling, stiffness/lock, and weakness; 2. Sports and daily activities – one item for sports and nine for daily activities; 3. Current knee function and knee function before injury – one item, not included in the total score composition [23]. Item 6 dichotomizes the answer into yes/no; items 1, 4, 5, 7, 8, and 9 use five-point Likert scales; and items 2, 3, and 10 are scored on an 11-point scale. Higher scores characterize greater commitment [23].

The Central Sensitization Inventory (CSI), through self-report, quantifies the degree of somatic and emotional complaints associated with central sensitization. Validated for the Brazilian population showing reliability (ICC > 0.80) and internal consistency (Cronbach’s α = 0.91). It is subdivided into two parts. A (25 items), in which each item can be scored on a Likert scale ranging from 0 to 4 points associated with the words “never” and “always”; and part B, a list of previous diagnoses related to central sensitization conditions. Severity levels are quantified in scores from 0 to 100, higher scores represent higher levels of central sensitization [24].

The 30 sec sit and stand test (30SSST) was performed in order to assess lower limb strength and endurance. For this, the same chair was used for all evaluations. The chair was placed against the wall to keep it from moving. Participants were instructed to sit in the middle of the chair, with their backs straight and their feet shoulder-width apart. The hands were placed on the opposite shoulder crossed at the wrists. Upon the command “Go”, the subject stood to a full standing position and then sat down again. In this way, the number of repetitions that the participant performed in 30 seconds was recorded [25].

### Statistical analysis

Histograms were created to determine the normality of the data. The Kolmogorov-Smirnov test was used to determine the normality of the data and the normal distribution of the variables was demonstrated. Pearson’s (r) and determination (R^2^) correlation coefficients were calculated to determine the strength of associations between variables. The magnitude of correlations was determined based on the classification proposed by Zou et al. [17]: 0 = no correlation, 0 ≥ 0.20 = weak correlation, 0.20 ≥ 0.50 = moderate correlation, 0.50 ≥ 0.80 = strong correlation, and 0.80 ≥ 1.00 = perfect correlation. All data were processed using the Statistical Package for the Social Sciences, version 17.0 (SPSS Inc., Chicago, Illinois).

## Results

**Fig. 1:**
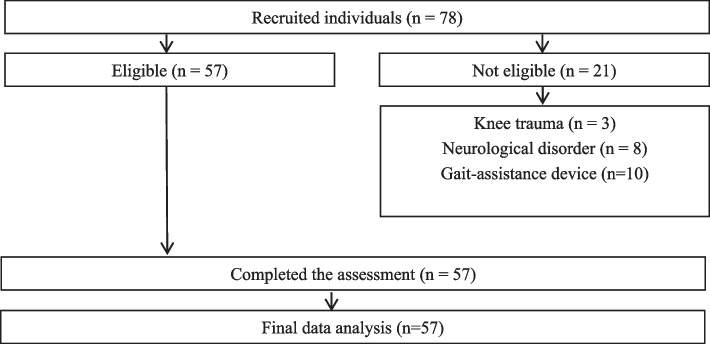
Flowchart of participants

A total of 78 individuals, with knee osteoarthritis, were recruited for the study, of these, 21 were excluded, based on the eligibility criteria; thus, the final sample consisted of 57 individuals (Fig. 1).

Table 1 displays the demographic characteristics and clinical variables of the individuals included in the present study. Participants were 94.73% female, with a mean age of 64 years and BMI of 25.9 Kg/m2 and 50.9% had a diagnosis of KOA in the left knee.

**Table 1:** Demographic characteristics and clinical variables of individuals with knee osteoarthritis (*n* = 57)

Variables	Mean (standard deviation) or number (percentage)
Body mass (kg)	65,7 (12,5)
Height (m)	1,59 (0,08)
BMI (kg/m^2^)	25,9 (4,6)
Age (years)	64 (5)
Sex - Male	3 (5,27%)
Sex - Female	54 (94,73%)
Affected side - left	29 (50,9%)
Affected side - right	19 (33,3%)
Affected side - bilateral	9 (15,8%)
CSI (score)	25,6 (3,4)
NRPS - R (score)	4,1 ± 1,0
NRPS - M (score)	4,8 ± 0,8
IKDC (score)	40,5 ± 3,8
30SSST (repetitions)	10,7 ± 1,4
EARS (score)	12,72 ± 2,0
EARS-RA (score)	19,3 ± 2,3

*BMI* Body mass index, *CSI* Central Sensitization Inventory, *NRPS-R* Numerical rating pain scale in rest, *NRPS-M* Numerical rating pain scale post movement, *IKDC* The International Knee Documentation Committee, *30SSST* 30 sec sit and stand test, *EARS* Exercise Adherence Rating Scale, *EARS-RA* Exercise Adherence Rating Scale - reasons for adherence

Table 2 shows the correlations between adherence behavior, central sensitization symptoms, the intensity of pain on rest and movement, knee disability symptoms, and functionality with no significant association between variables.

**Table 2:** Correlation between EARS and clinical variables of individuals with knee osteoarthritis (*n* = 57)

Variables	EARS		
	R	R^**2**^	***p***
CSI (score)	-0,005	< 0,0001	0,971
NRPS - R (score)	-0,091	0,0082	0,502
NRPS - M (score)	0,106	0,0112	0,431
IKDC (score)	0,144	0,0207	0,286
30SSST (repetitions)	-0,114	0,0129	0,398

*CSI* Central Sensitization Inventory, *NRPS-R* Numerical rating pain scale in rest, *NRPS-M* Numerical rating pain scale post movement, *IKDC* The International Knee Documentation Committee, *30SSST* 30 sec sit and stand test, *EARS* Exercise Adherence Rating Scale

Table 3 shows the correlations between reasons for adherence, central sensitization symptoms, the intensity of pain on rest and movement, knee disability symptoms, and functionality with no significant association between variables.

**Table 3:** Correlation between EARS-RA and clinical variables of individuals with knee osteoarthritis (*n* = 57)

	EARS-RA		
Variables	R	R^**2**^	***p***
CSI (score)	0,110	0,0121	0,415
NRPS - R (score)	0,031	0,0009	0,816
NRPS - M (score)	0,067	0,0044	0,619
IKDC (score)	0,136	0,0184	0,313
30SSST (repetitions)	0,116	0,0134	0,389

*CSI* Central Sensitization Inventory, *NRPS-R* Numerical rating pain scale in rest, *NRPS-M* Numerical rating pain scale post movement, *IKDC* The International Knee Documentation Committee, *30SSST* 30 sec sit and stand test, *EARS-RA* Exercise Adherence Rating Scale - reasons for adherence

## Discussion

The present study identified no significant association between adherence to a home exercise program and central sensitization, pain intensity, and anxiety in functionality with KOA. Our hypothesis was that variables related to functionality and pain would be positively associated with adherence to a home exercise program for individuals with KOA.

It is currently understood that patient characteristics and medical history, including clinical variables and knowledge about prevention, are dependent variables in relation to adherence to exercises [26]. These conclusions are supported by prediction models used to assess adherence to exercise therapy in individuals with KOA [26]. In the same way as variables related to motivation levels, socioeconomic status, beliefs, goals, and personal values are routinely associated with the ability to adhere to exercise in individuals with osteoarthritis [5, 9, 11].

However, these results are related to exercise adherence in general. No specific analysis related to home exercise program protocols for individuals with KOA. The fact is that even having carried out a cross-sectional study, limits the proof of causality of the associations. Our study differs from the others by specifying the type of exercise related to adherence. And even more, understanding that signs and symptoms related to pain and disability are highlighted as potential influencers for adherence to exercises in different clinical conditions [5]. We use clinical and validated instruments to perform a broad analysis of variables related to pain and disability with adherence to the home exercise program in individuals with KOA.

Despite different methodologies and the fact that we used a valid and specific instrument to analyze adherence to home exercises. Our results complement the findings of Vries et al. [27], who carried out a convergent mixed methods study (qualitative and quantitative analysis), attesting that intrinsic factors involving pain and functionality are not related to the behavior of adherence to home exercises. Vries et al. [27], also suggest that, when it comes to exercises performed at home, the physical therapist exerts a positive influence on adherence, mainly through the customization of the program to be applied and the participant’s motivation.

Our results do not define the causality of associations between variables related to pain and functionality with adherence to a home exercise program. However, the analysis of this association serves to alert clinicians and researchers to be aware that variables related to pain and functionality do not seem to be associated with the adherence of individuals with KOA to home exercise protocols. Perhaps, our results point to the need to carry out longer, more robust future studies, with specific assessment instruments and focused on the behavior of participants and therapists [27, 28]. Especially when we analyze the results highlighted by Jouper et al. [28], where they indicated that health professionals should strengthen the individual’s intention to exercise, allowing a calm state of energy before the beginning of the exercise and stimulating concentration during the performance of the exercises are fundamental.

Specifically on the level of adherence found in our results, even using a home exercise protocol characterized by promoting a reduction in pain intensity, and joint stiffness, increasing muscle strength of the lower limbs and promoting improved balance and mobility [16]. Our participants did not reach 17/24 points on the EARS, defined as an acceptable cut-off point for adherence [21]. However, two other studies [14, 29] with longer follow-ups and composed of participants with characteristics like our study also did not reach this cut-off point.

The issue of adherence to home exercises is complex and with several nuances for clinicians and researchers. However, both must bear in mind that in addition to increased adherence to exercises, it does not directly translate into improved pain and functionality [14]. Apparently, the variables central sensitization, pain intensity, and functionality are not associated with adherence to a home exercise program for individuals with KOA. We believe that our results do not define causal associations, nor do they directly change clinical strategies. However, they open the possibility for further studies to be carried out. Mainly, prioritizing not only the analysis of variables linked to pain or function. And yes, of variables not analyzed in this study. Focused on behavioral changes, awareness, and understanding of the disease by the individual diagnosed with KOA and therapist-related behaviors [30].

Several limitations are acknowledged. First, as this is a cross-sectional study, the results presented here must be interpreted with caution. For it does not define the causality of associations. It only analyzes the association between adherence to a home exercise program and central sensitization, pain intensity, and functionality in individuals with KOA. A maximum limit was defined in relation to the age of the participants, a fact that may limit the overlapping of the results for age groups not covered. Still, on the characteristics of the study participants, radiographic confirmation, and Grades 2 or 3 of the Kellgren-Lawrence Classification were used. Even though they are classically recommended [18], they may limit the extrapolations of results to grade 4. Participants had already participated in activities related to the pain care group. Therefore, they may have been more motivated to participate in the study. This characteristic may not be replicated for other groups involving individuals with KOA. As it is an already structured care group, we did not interfere in the composition and planning of the therapeutic plan carried out. Finally, data were collected during the second half of 2021. During this period, some government measures to combat COVID-19 were in effect, which may have influenced the participation of individuals.

## Conclusion

No significant association was identified between adherence to a home exercise program and central sensitization, pain intensity, and functionality in individuals with KOA.

## Data Availability

The datasets generated and/or analyzed during the current study are not publicly available due to our limitation of digital data stores for collective access but are available from the corresponding author on reasonable request.
